# Supplementary material for: Content-rich biological network constructed by mining PubMed abstracts
Source: BMC Bioinformatics. 2004 Oct 8;5:147. doi: 10.1186/1471-2105-5-147 (PMC528731; doi:10.1186/1471-2105-5-147)
Supplement: Additional File 2 — The original results of the above study (non-essential files are deleted to keep the file size under the limit set by BMC bioinformatics). [file 1471-2105-5-147-S2.bz2 › chilibotAdditionalFile2/dip10/620ID8598903E11005/html/F7_F3.html]

 


 **F7** and **F3** 
  
Found 158 abstracts in PubMed, retrieved 10.  
 

 What does Google say? 
 PDF only 
| .edu only 

---

**Interactive relationship** (e.g. stimulation, inhibition, etc)

**Neutral relationship**- Tissue factor  [ **F3** ]  TF is the cellular receptor and cofactor for blood coagulation factor VII  [ **F7** ]  FVII.  Ref: 12823950 Trends Pharmacol Sci, 2003

**Non-interactive relationship** (e.g. studied together, co-existance, homology, etc.)

- Tissue factor  [ **F3** ]  TF, a cell surface associated cofactor and activator of coagulation factor VII  [ **F7** ] , has been implicated in the local and systemic activation of coagulation associated with sepsis.  Ref: 12658585 Vet Clin Pathol< MedlineTA>Vet Clin Pathol, 2003
- Increased SCD was observed at left frontotemporal FT3, bilateral temporoparietal TP3 and TP4 and prefrontal locations F8 F4 and  **F7**   **F3** , suggesting novelty P3 generators located in the left auditory cortex, and bilaterally in temporoparietal and prefrontal association regions.  Ref: 12706218 Brain Res Cogn Brain Res, 2003
- In such women the arterial intima can express tissue factor  [ **F3** ] , and changes in coagulation factor VII  [ **F7** ]  factor VII and tissue factor  [ **F3** ]  pathway inhibitor TFPI may be deleterious.  Ref: 12456630 Hum Reprod, 2002
- We found that only fractions  **F3**  and  **F7**  inhibit the tested enzyme.  Ref: 12490243 Fitoterapia, 2002
- Effects of different progestin regimens in hormone replacement therapy on blood coagulation factor VII  [ **F7** ]  and tissue factor  [ **F3** ]  pathway inhibitor.  Ref: 12456630 Hum Reprod, 2002
- After varimax rotation, we obtained seven independent factors F1 Disinhibition instability, F2 Paranoia hostility,  **F3**  Deficit, F4 Grandiosity psychosis, F5 Elation euphoria, F6 Depression, and  **F7**  Hyper sexuality.  Ref: 12507733 J Affect Disord, 2003
